# Associations Between Gender and Obesity Among Adults with Mental Illnesses in a Community Health Screening Study

**DOI:** 10.1007/s10597-015-9965-2

**Published:** 2015-12-28

**Authors:** Jessica A. Jonikas, Judith A. Cook, Lisa A. Razzano, Pamela J. Steigman, Marie M. Hamilton, Margaret A. Swarbrick, Alberto Santos

**Affiliations:** Department of Psychiatry, Center on Mental Health Services Research and Policy, University of Illinois at Chicago, 1601 West Taylor Street, M/C 912, Chicago, IL 60612 USA; Collaborative Support Programs of New Jersey, Department of Psychiatric Rehabilitation and Counseling Professions, Rutgers, The State University of New Jersey, 8 Spring Street, Freehold, NJ 07728 USA; Department of Psychiatry and Health Behavior, Georgia Regents University, 1120 15th Street, Augusta, GA 30912 USA

**Keywords:** Obesity and gender, Serious mental illness, Health disparities, Public mental health

## Abstract

The prevalence of obesity and its associations with gender, clinical factors, and medical co-morbidities were examined among 457 adults attending public mental health programs in 4 U.S. states. BMI was measured directly and other information was gathered by interview. Over half (59 %, n = 270) were obese including 18 % (n = 83) who were morbidly obese. In hierarchical ordinary least squares regression analysis controlling for demographic, psychiatric, medical, smoking, and health insurance statuses, women were significantly more likely to be obese than men. Obesity also was more likely among those who were younger and not high school graduates, those with diabetes or hypertension, and those who did not smoke tobacco. Interaction effects were found between gender and diabetes, hypertension, tobacco smoking, education, race, and age. The high prevalence of obesity among women, coupled with interactions between gender and other factors, suggest that targeted approaches are needed to promote optimal physical health in this population.

## Introduction

Current estimates suggest that nearly one-third of the world’s population is overweight or obese, with obesity now characterized as a global health crisis (Low et al. 2009). The number of overweight and obese individuals worldwide has increased from 857 million (20 %) in 1980 to 2.1 billion (30 %) in 2013 (Ng et al. 2014). Among higher income countries, some of the largest increases in adult obesity have occurred in the United States (one-third of adults), Australia (nearly 30 %), and the United Kingdom (around 25 %) (Ng et al. 2014). Globally, a higher proportion of women are obese than men (Low et al. 2009), yet most obesity treatment and service options are not gender-informed (Kanter and Caballero 2012). Obesity, especially morbid obesity, is a risk factor for numerous medical conditions, including diabetes mellitus, hypertension, cardiovascular disease, metabolic syndrome, and some cancers (Mokdad et al. 2003).

Current research suggests that overweight and obesity are more prevalent among adults with serious mental illnesses than those in the general population (Bradshaw and Mairs 2014; Compton et al. 2006). People with mental disorders also are more vulnerable to the health risks associated with obesity such as type 2 diabetes, heart disease, stroke, high blood pressure, high cholesterol, and premature death (De Hert et al. 2011). Factors associated with obesity for people with mental illnesses include use of psychotropic medication (Virk et al. 2004), poverty (Glover et al. 2013), lack of physical activity and exercise (Daumit et al. 2005), high volume of food intake (McKibbin and Kitchen 2014; Strassnig et al. 2003), and diets high in fat and low in fiber (Casagrande et al. 2011; Brown et al. 1999).

Several studies suggest that the prevalence of obesity is higher among female psychiatric outpatients than male psychiatric outpatients. For example, Kolotkin et al. (2008) studied adults with schizophrenia and bipolar disorders recruited from four outpatient treatment programs. They found that obese participants were significantly more likely to be women (59 %) than men (44 %), using Fisher’s exact test.

Coodin (2001) compared the Body Mass Index (BMI) scores of a clinic-based sample of adults with schizophrenia to those in the general Canadian population. Among the four groups, only women with schizophrenia had a mean BMI in the obese range (30.0), compared to men with schizophrenia (28.5), men without schizophrenia (26.3), and women without schizophrenia (24.3). These results were confirmed by a study that compared randomly selected U.S. psychiatric outpatients with matched individuals from the National Health and Nutrition Examination Survey (NHANES) III (Dickerson et al. 2006). Controlling for age, race, and smoking status, a mean adjusted BMI score in the obese range was found only for the female psychiatric sample (32.3), but not their male counterparts (29.0), or the matched females (27.2) and males (26.8).

Allison et al. (1999) used National Health Interview Survey (NHIS) data to compare BMIs among men and women with schizophrenia, and men and women without schizophrenia. They found that women with schizophrenia had significantly higher BMIs than female respondents without schizophrenia, controlling for age (mean BMI of 27.36 vs. 24.50, respectively). In contrast, there was no significant difference between the BMIs of men with and without schizophrenia (mean BMI of 26.14 vs. 25.63, respectively). The authors suggested that women with schizophrenia are overrepresented among the obese compared to the other three groups.

In their study of randomly sampled public health insurance recipients in the state of Maryland, Daumit et al. (2003) found that the prevalence of obesity was two times greater among female outpatients than male outpatients, even after adjusting for age, race/ethnicity, and use of tobacco. Comparing this group to a matched sample of men and women who participated in the NHANES III, approximately 81 % of the women outpatients were overweight or obese compared to 56.3 % of matched NHANES females. Among males, however, the proportions were highly similar (61 vs. 56.3 %, respectively).

One final study’s findings regarding gender and obesity were more equivocal. In research on adult outpatients with bipolar disorder participating in an international treatment outcomes study (McElroy et al. 2002), male outpatients had significantly higher rates of overweight than females, no gender differences were found in proportions obese, and female outpatients had significantly higher rates of morbid obesity than males. However, in a multivariate analysis, only male gender was associated with a greater likelihood of being overweight, and sex was not significant in the models predicting obesity or morbid obesity.

Less attention has been paid to how race and ethnicity impact the likelihood of obesity for people with mental illnesses (Carliner et al. 2014). While two studies found no statistically significant differences in the prevalence of overweight or obesity by race/ethnicity among adult outpatients (Daumit et al. 2003; Kolotkin et al. 2008), two others found significantly higher obesity among African American versus white outpatients (Kemp et al. 2014; Schneiderhan et al. 2009). Even less research attention has been devoted to how gender interacts with race to impact on obesity and overweight (Carliner et al. 2014). One study found that African American female psychiatric outpatients had significantly higher BMIs than white female psychiatric outpatients (Strassnig et al. 2011), while another found that Hispanic male outpatients had higher BMIs than non-Hispanic males receiving outpatient treatment (McEvoy et al. 2005).

Age also has been found to be associated with obesity among people with serious mental illnesses, a finding that may vary by gender. For example, one study found that older age confers greater odds for obesity among female outpatients, while increasing age in male outpatients is associated with lesser likelihood of obesity (Daumit et al. 2003). A final demographic variable, education, has been found to be associated with obesity in the general population, with lower education linked to a higher prevalence of obesity (Schnohr et al. 2004). However, little research attention has been paid to this association among adults with mental illnesses.

Recent estimates suggest that users of public health insurance (e.g., Medicaid and Medicare) have a higher prevalence of obesity than those who are uninsured and privately insured. For example, Finkelstein et al. (2003) combined NHIS with Medical Expenditure Panel Survey data and found that the U.S. prevalence of obesity was 27 % for Medicaid users, 19 % for Medicare, 17 % for private, and 17 % for uninsured respondents. Given that many public mental health clients are Medicaid or Medicare beneficiaries (Rowan et al. 2013), insurance coverage is an important factor to consider when examining the prevalence of obesity among adults with mental illnesses. Also associated with obesity for people with mental illnesses is the presence of co-morbid diabetes and hypertension (Dickerson et al. 2006; McElroy et al. 2002). Some have suggested that residential status also is associated with obesity among adult psychiatric outpatients, given that many live in congregate settings where nutrition is often poor and fiscal constraints promote high calorie, high carbohydrate diets (Wallace and Tennant 1998). Even if they do not reside in congregate housing, many people in mental health recovery live in “food deserts,” or impoverished regions with poor access to fresh food, in which the rates of obesity are higher than for those living in wealthier communities (Levine 2011).

Finally, studies of the general population suggest that tobacco use has differential impact on weight, with light to moderate smokers being less likely to be overweight and heavy smokers more likely to be obese (Chiolero et al. 2008). Given that individuals with serious mental illnesses are significantly more likely than the general population to be both obese and smokers (Ferron et al. 2011), this relationship should be considered when examining weight among psychiatric outpatients.

While a burgeoning literature suggests that the impact of obesity on adults with mental illnesses is an important public health issue, additional research is needed to establish within-population gender differences in obesity among diagnostically heterogeneous psychiatric outpatients, which is the focus of this analysis. Also of importance is whether and how race and ethnicity, age, level of education, residential status, health insurance, and use of tobacco affect the impact that gender may have on obesity in this population. Our first hypothesis was that the likelihood of obesity would be greater among women than men attending community mental health programs for individuals with serious mental illnesses. Our second hypothesis was that this relationship would persist despite controlling for demographic characteristics, systemic factors, and medical co-morbidities found in prior studies to influence the likelihood of obesity.

## Methods

### Participants

Health screenings of adults served in publicly-funded community mental health programs in four U.S. states were conducted via 3-day health fairs designed and managed by a university research center and a mental health peer-run collaborative (Swarbrick et al. 2013). The first screening was held at a peer-operated mental health program in New Jersey, and attended by members of peer-run self-help centers across the state. The second took place on a university campus in Illinois, and was attended by members of a city-wide psychiatric rehabilitation agency. The third was held in two community venues, and attended by clients of mental health agencies in Maryland. The fourth occurred at a community mental health agency located in Georgia. Participants were recruited by program staff through clinician referral, flyers, announcements at program meetings, waiting room posters, and word-of-mouth. Only 2 % (7 of 464) of those attending the screenings refused participation. Written informed consent was obtained from participants on the day of the screening, using procedures approved by the University of Illinois at Chicago Institutional Review Board (IRB). There were no known conflicts of interest among the study’s authors, and all certify their responsibility for this manuscript.

Eligibility criteria included serious mental illness as defined by U.S. federal Public Law 102–321 to include a *DSM*-*IV*-*TR* diagnosis of mental illness accompanied by moderate to severe functional impairment; age 18 years or older; English speaking; status as a client of the participating community mental health program; and ability to provide informed consent.

### Procedures

Following completion of the informed consent process, a standardized research protocol was administered by trained research interviewers, using laptop computer-assisted software that included built-in skip logic and consistency checks. Participants next visited screening stations at which the following measures were taken or tests were administered: BMI; blood pressure; blood glucose profile; non-fasting lipid profile; risk for alcohol and drug abuse; nicotine use and dependence; and risk for coronary heart disease. Screening staff included medical students, psychiatry residents, researchers, and peer specialists, each of whom completed a minimum of 6 hours of training on methods for administering each test, including observation and corrective feedback. All serologic testing was conducted by licensed nurses or other medically trained staff. At the final station, participants met with peer health and wellness specialists who explained test results, answered questions, and offered strategies for health promotion (Swarbrick et al. 2013). A research honorarium was provided in the form of a gift card from a local store as well as a gift bag containing wellness products. Participants also were given a list of free local health care clinics at which they could follow-up on their screening results if they lacked a primary care provider. They also had opportunities to visit health promotion and education booths at the fair.

### Measures

#### Body Mass Index

Body Mass Index (BMI, kg/m^2^) measurements were taken directly from participants who wore lightweight indoor clothing and shoes during assessment. Weight was measured using a portable, medical-grade, digital scale that was recalibrated at each study site. Height was measured via wall chart. Weight was recorded to the nearest pound, and height to the nearest half-inch.

#### Co-morbidities and Background Characteristics

Prevalence of common medical conditions, including diabetes and hypertension, was assessed using items from the National Health and Nutrition Examination Survey (NHANES) (CDC 2010). Respondents were asked whether they had ever been told by a doctor or other health professional that they had specific medical conditions and, if so, whether they still had the condition and were in treatment for it. Tobacco use was assessed using the Fagerstrom Test for Nicotine Dependence (Heatherton et al. 1991), in which individuals respond to items related to the amount and frequency of their use of nicotine. Also assessed were demographic characteristics (e.g., sex, race, ethnicity, age, education, residential status), psychiatric diagnosis, and health insurance status and type.

### Statistical Methods

Descriptive statistics were calculated for all model variables and participant background characteristics. To evaluate our first hypothesis we used Chi square and the *t* test statistic to calculate associations between gender and BMI score, ordinal categories of BMI (i.e., underweight, normal, overweight, obese, morbidly obese), and our dichotomous dependent variable, defined as obese = 1/non-obese = 0. To evaluate our second hypothesis, multivariable logistic regressions analysis (Freedman 2009) was used to test associations between obesity and model variables by entering different domains sequentially in the following hierarchical steps: (1) demographic characteristics (gender, race, residential status, age, education); (2) insurance status (Medicaid, Medicare, or private health insurance coverage versus none); (3) clinical factors (diagnosis of schizophrenia, mood disorder); (4) medical co-morbidities (diabetes, hypertension); and (5) smoking status. Also included were 3 dichotomous variables representing study site (i.e., NJ, MD, GA), with the 4th site (IL) omitted and used as a contrast. Finally, we used logistic regression analysis to test for two-way interaction effects between gender and each factor found to be significant in the multivariable analysis. This was done by classifying participants into 4 groups by gender and the presence or absence of each factor (e.g., women smokers, women non-smokers, men smokers, men non-smokers), and then including dichotomous variables for 3 of the groups in logistic regression analyses, omitting the 4th group comprised of men without the characteristic (e.g., men non-smokers) and using it as a contrast. All interaction analyses controlled for study site to ensure that any significant interactions were not confounded by geographical region. Absence of multicollinearity was confirmed by the fact that none of the model variables had zero-order correlations r ≥ |.5|.

## Results

### Background Characteristics and Representativeness

Table 1 presents participants’ demographic and clinical characteristics. Comparison of these characteristics to those of adult populations at each agency (not shown) revealed no significant differences by sex, race, Hispanic/Latino ethnicity, education, age, diagnosis, and health insurance status with two exceptions. At the NJ site, a smaller proportion of males were screened (46 %) than in the agency population (59 %) (Z = −2.1, *p* < .05). At the GA site, more screening participants reported a diagnosis of schizophrenia (40 %) than the agency population (25 %) (Z = 3.3, *p* < .001). Otherwise, screening participants were representative of the agency populations targeted.

**Table 1:** Characteristics of adults with serious mental illness screened for Body Mass Index (N = 457)

	Total^a^	
	N	%
Sex		
Female	221	48.4
Male	236	51.6
Race		
White	221	48.8
Black/African American	175	38.6
Asian/Pacific Islander	7	1.5
American Indian/Alaskan Native	2	.4
Multi-racial	17	3.8
Other	30	6.6
Hispanic/Latino Ethnicity	32	7.1
Education		
<High school	89	20.0
High school/GED	138	31.1
Some college/advanced degree	217	48.9
Marital status		
Married/cohabiting	35	7.9
Never married	292	65.8
Widowed/separated/divorced	117	26.4
Mean (SD) age, years	46.5	(12.1)
Health insurance type		
Medicaid	130	29.2
Medicare	82	18.4
Dual	137	30.7
Private	43	9.7
Veteran’s	11	2.5
Other	22	5.2
None	62	13.9
*DSM*-*IV* diagnosis		
Schizophrenia	179	40.6
Bipolar disorder	100	22.7
Depression	106	24.0
Anxiety disorder	19	4.3
Personality disorder	4	.9
Other	33	7.5
Study site		
New Jersey	121	26.7
Illinois	122	26.8
Maryland	106	22.8
Georgia	108	23.7

^a^Variations in sample size are due to missing data

Of the 452 participants assessed, 2 % (n = 7) were underweight, 17 % (n = 75) were normal weight, 22 % (n = 100) were overweight, 41 % (187) were obese, and 18 % (n = 83) were morbidly obese. The mean (standard deviation = SD) BMI was 32.4 (SD = 8.4). Tests for differences by gender revealed that a higher proportion of men than women were under- or normal weight (60.5 vs. 39.5 %, respectively), a higher proportion of men than women were overweight (64.0 vs. 36.0 %, respectively), roughly equal proportions of men and women were obese (50.3 vs. 49.7 %, respectively), and more women than men were morbidly obese (71.1 vs. 28.9 %, respectively) (X^2^(3) = 25.92, *p* < .001). On average, BMI for women was significantly higher (34.2) than for men (30.6) (t(419) = 4.5, *p* < .001 (CI 2.0–5.1). Women also were significantly more likely to be obese or morbidly obese (68.9 %) than men (50.8) (X^2^(1) = 15.24, *p* < .001).

Table 2 presents the results of a stepped logistic regression model predicting the likelihood of obesity. Step 1 involved entering six demographic characteristics. Women were over two times as likely as men to be obese, and those living in supported or congregate residential settings were around two-thirds as likely to be obese compared to those living independently. There were no relationships between obesity and race, age, or education. In step 2, health insurance coverage was not significant, and the residential status variable became non-significant. In step 3, involving the addition of clinical factors, those with mood disorders were almost twice as likely to be obese as those with other diagnoses. In this step, the diagnosis of schizophrenia was not significant. In step 4, involving the addition of medical co-morbidities, people with diabetes were over two and one-half times as likely to be obese as those without diabetes, and those with hypertension were over one and one-half times as likely to be obese as those without high blood pressure. In this step, the age and education variables became significant, with younger participants more likely to be obese than older ones, and high school graduates half as likely to be obese as those without a high school or equivalency degree. In step 5, involving the addition of smoking status, individuals who smoked tobacco were around half as likely to be obese as non-smokers, and all previously significant variables remained significant. Thus, in the final step of the analysis, controlling for demographic, systemic, clinical, and medical co-morbidity factors, women with serious mental health conditions were almost twice as likely to be obese as men.

**Table 2:** Multivariable associations of obesity with health-related domains in hierarchical logistic regression models; all steps control for study site

	Step1	Step2	Step3	Step4	Step5
Step 1					
*Demographics*					
Female	2.28***	2.26***	2.16***	2.03***	1.94**
African American	1.74	1.78	1.84	1.74	1.71
Congregate residential status	.63*	.64	.64	.68	.74
Age	.99	.99	.99	.98*	.97**
High school graduate	.60	.60	.61	.52*	.46**
White	1.67	1.71	1.71	1.84	1.76
Step 2					
*Systemic factors*					
Any health insurance		1.54	1.55	1.51	1.35
Step 3					
*Clinical factors*					
Mood disorder			1.88*	1.60	1.76
Schizophrenia			1.39	1.24	1.31
Step 4					
*Co*-*morbidities*					
Diabetes				2.72***	2.82***
Hypertension				1.62*	1.64*
Step 5					
*Tobacco use*					
Smokes tobacco					.45***

*** *p* < .001; ** *p* < .01; * *p* < .05

Next, we tested for interaction effects between gender and all variables that remained significant in the final step of our multivariable logistic regression analysis. We stratified participants into 4 groups by gender and the presence or absence of each characteristic, and used men without the characteristic as the contrast in logistic regression analyses controlling for study site. As shown in Table 3, we found significant interaction effects between female gender and diabetes, hypertension, smoking, and education, and between male gender and age, smoking, and diabetes. Here, women with diabetes were over 5 times as likely to be obese as men without diabetes. Women with hypertension were over 3 times as likely to be obese as men without hypertension. Women non-smokers were over 2 and one-half times as likely to be obese as male non-smokers. Women without a high school degree were over 3 and one-half times as likely to be obese as males who were not high school graduates. We found that men with diabetes were over 3 times as likely to be obese as men without diabetes. Men who smoked were half as likely to be obese as male non-smokers. Finally, older men were half as likely to be obese as younger men.

**Table 3:** Interaction effects of gender and significant model variables in associations with obesity, controlling for study site

	Diabetes	Hypertension	Tobacco smoker	High school graduate	Older age
Female, with^a^	5.57***	3.40***	.82	1.10	.96
Female, without^a^	2.02**	1.71^+^	2.60**	3.60**	2.20^+^
Male, with^a^	3.10**	1.40	.56*	.63	.50*

*** *p* < .001; ** *p* < .01; * *p* < .05; ^+^ *p* < .10

^a^Compared to Male, without

One final step in the analysis involved testing for interactions between gender and specific racial and ethnic minority statuses in predicting the likelihood of obesity. Results (not shown) revealed a significant interaction between race and gender in which African American women were twice as likely to be obese as non-African American women, and men of both races.

## Discussion

The results of our analysis confirmed our first hypothesis that the BMIs of women with serious mental illnesses would be more likely to be in the obese range than those of men with these illnesses. Confirming our second hypothesis, this relationship persisted despite controlling for other demographic, systemic, clinical, chronic illness, and smoking factors. Previous research suggests several possible reasons for this gender disparity, one being that physical inactivity is more prevalent among women than men psychiatric outpatients (Azarbad and Gonder-Frederick 2010). Another factor affecting greater likelihood of obesity among women is female weight gain related to hormonal transitions (Azarbad and Gonder-Frederick 2010). Further, childhood trauma, which has been found to be associated with obesity in women (Alvarez et al. 2007; Smith et al. 2010), is significantly more prevalent among women with mental illnesses than their male counterparts (Gearon et al. 2004; Perese and Perese 2003).

Consistent with previous research, we also found that younger participants, those who did not graduate from high school, those with diabetes, and those with hypertension were more likely to be obese. Moreover, interaction effects were found for gender with diabetes and hypertension. Specifically, women with diabetes or hypertension, and men with hypertension were more likely to be obese than their counterparts. This suggests that weight management programs should be tailored for persons with these chronic medical conditions (Green et al. 2014), including those promoting diets that foster insulin control concurrent with weight loss (Parker et al. 2002), and physical activities appropriate for those with both controlled and uncontrolled high blood pressure (Bacon et al. 2004). We also found an interaction effect between gender and education, where women with less formal education were more likely to be obese. This suggests that weight management interventions should be designed at appropriate levels of reading ability and also for those with low levels of health literacy that typically is related to lower levels of formal education (Brucki et al. 2011).

We found a strong association between smoking and a lower likelihood of obesity, suggesting that some women may use smoking to control their weight, either consciously or unconsciously. If this is so, smoking cessation interventions should be tailored to take account of the potential for weight gain among women who are trying to quit or reduce their dependence on nicotine. Given that women express greater concerns than men about weight gain following cessation attempts (Pomerleau and Saules 2007), gender-sensitive approaches are needed, such as those using cognitive behavioral therapy to address women’s weight concerns as part of smoking cessation treatment (Perkins et al. 2001). Efforts also should be made to prevent relapse among those women who have quit smoking but then encounter distressing weight gains. Our results suggest that nicotine replacement therapy and supportive counseling alone may not be adequate to address these interlocking issues.

In the final step of our model, there were no significant associations between mental health diagnosis and the likelihood of obesity. This mirrors the results of prior studies that also failed to find associations between obesity or being overweight and clinical factors such as diagnostic subtypes, number of psychiatric episodes, or age at onset of mental illness (Kolotkin et al. 2008; McElroy et al. 2002).

We did not find statistically significant racial differences in obesity prevalence in our stepped regression analysis, similar to the findings of some prior studies (Daumit et al. 2003; Keenan et al. 2013). Perhaps the racial disparity in obesity typically observed in the general U.S. population (Wang and Beydoun 2007) was masked by psychotropic medication-induced weight gain often experienced by psychiatric outpatients (Virk et al. 2004). Other possibilities are that racial and ethnic disparities were moderated by constraints on exercise due to poor health (Glover et al. 2013), unhealthy eating patterns (Carson et al. 2015), or low health literacy regarding nutritional and food information (Lincoln et al. 2008). However, as in other studies linking minority status, obesity, and poor mental health (Hicken et al. 2013; Rosen-Reynosoa et al. 2011), we did find an interaction between race and gender in which African American women were twice as likely to be obese as non-African American women and men of both races. This underscores the need to incorporate cultural elements that address the needs and strengths of black women’s weight management, such as teaching them how to alter their traditional recipes to be lower in salt and fat, and involving their family members directly in weight loss efforts (Ard et al. 2000).

While gender-informed health interventions are necessary, they may not be sufficient to address weight management for women outpatients. As suggested by Pederson et al. (2015), gender-transformative efforts also could encompass emerging evidence on women’s unique challenges in achieving healthy lifestyles. Such endeavors would attend to the structural and policy changes needed to mitigate the weight-impacting stressors that women with mental illnesses experience, including multiple forms of discrimination, parenting in difficult circumstances, and managing in resource-poor environments that make healthier lifestyles more challenging to achieve (Cook and Mueser 2013). Also considered should be how opportunities for exercise often reflect the norms of a male-centered sport and recreation culture that can be daunting to many women (Liwander et al. 2013), and how implementing trauma-informed policies would promote health without shaming or stereotyping women for their body size regardless of their weight (Pederson et al. 2015).

Our study had several limitations. First, participants were recruited from selected community mental health programs in four U.S. states and were not representative of adults with serious mental illnesses. Second, these treatment programs may have been more or less attentive to the physical health of clients than other agencies, and thus, not representative of community mental health programs generally. Third, study participants were self-selected, and may not have represented their larger agency populations (although statistical tests confirmed their representativeness on a number of background variables). Fourth, we were unable to assess other important variables associated with overweight and obesity, and whether these varied by gender, including family history, dietary habits, experience of trauma, or level of physical activity. Fifth, we did not obtain data on use of psychiatric or other medications, particularly those that have been found to be associated with weight gain, such as atypical antipsychotics and antidepressants. However, as other investigators have noted (Daumit et al. 2003), the complexity of most psychiatric medication regimens, combined with the relatively small number of patients on each specific regimen, would most likely have precluded our ability to measure any associations. Nonetheless, it bears noting that women are more likely than men to experience weight-related medication side effects. Several studies have shown, for example, that the weight gain associated with antipsychotic medications is significantly higher among women than men (Covell et al. 2004; Homel et al. 2002). Because women have more fatty tissue than men, antipsychotic drug levels build up in women’s bodies, leading them to report significantly more side effects, including weight gain, than do their male counterparts (Seeman 2010). To make matters more complicated, when a woman who has used antipsychotics diets and loses body fat, higher concentrations of medication previously stored in the fat compartment are released into her bloodstream, which can result in increased side effects (Seeman 2010). These complex issues call for the development of interventions that apply shared decision-making principles and strategies to support women in making informed decisions about the potential advantages and disadvantages inherent in psychiatric medications, especially those that lead to the weight gain associated with many serious medical conditions. Additionally, adherence to recommended clinical guidelines when prescribing psychiatric medications might also reduce weight-related side effects. These guidelines include: (1) a baseline assessment of BMI and any metabolic risk factors when patients start new psychiatric medications; (2) regular and ongoing monitoring of patients for signs of metabolic risk factors that could lead to changing a medication and/or a referral to specialized services; and (3) encouraging patients to track and report any alteration in their weight following a medication change (American Diabetes Association et al. 2004).

Strengths of our study include the use of a diagnostically heterogeneous outpatient population, offering a more complete picture of associations between gender and obesity, as well as other important covariates, across different diagnostic categories (Razzano et al. 2015). Another strength is our use of measured weight and height to calculate BMI, rather than self-report, given considerable evidence that adults underestimate weight and overestimate height (Pursey et al. 2014), and the fact that this tendency is worse among women than men (Engstrom et al. 2003) and obese than non-obese individuals (Gosse 2014). A third strength is the multi-site nature of our design, given that we were able to include programs in the Southern, Midwestern, and East Coast regions of the U.S. To our knowledge, our study represents the first analysis of within-population gender differences in obesity and weight-related conditions among diagnostically heterogeneous psychiatric outpatients in multiple geographic areas, which has implications for designing and implementing gender-informed services.

In conclusion, we documented the differential impact of gender on the likelihood of obesity, and interrelations between gender and important co-variates including age, education, smoking, diabetes, and high blood pressure. Future research is needed on how obesity differentially affects women and men with psychiatric disorders, especially when using medications, and how best to tailor treatment and support services for gender-specific needs.
